# Characterization of Microcrack Orientation Using the Directivity of Secondary Sound Source Induced by an Incident Ultrasonic Transverse Wave

**DOI:** 10.3390/ma13153318

**Published:** 2020-07-25

**Authors:** Jishuo Wang, Caibin Xu, Youxuan Zhao, Ning Hu, Mingxi Deng

**Affiliations:** 1 College of Aerospace Engineering, Chongqing University, Chongqing 400044, China; WangJishuo1224@163.com (J.W.); caibinxu0839@cqu.edu.cn (C.X.); youxuan.zhao@cqu.edu.cn (Y.Z.); 2 School of Mechanical Engineering, Hebei University of Technology, Tianjin 300401, China; ninghu@hebut.edu.cn

**Keywords:** ultrasonic transverse wave, microcrack orientation, bilinear stress–strain model, secondary sound source, directivity

## Abstract

In this paper, characterization of the orientation of a microcrack is quantitatively investigated using the directivity of second harmonic radiated by the secondary sound source (SSS) induced by the nonlinear interaction between an incident ultrasonic transverse wave (UTW) and a microcrack. To this end, a two-dimensional finite element (FE) model is established based on the bilinear stress–strain constitutive relation. Under the modulation of contact acoustic nonlinearity (CAN) to the incident UTW impinging on the microcrack examined, the microcrack itself is treated as a SSS radiating the second harmonic. Thus, the directivity of the second harmonic radiated by the SSS is inherently related to the microcrack itself, including its orientation. Furthermore, the effects of the stiffness difference between the compressive and tensile phases in the bilinear stress–strain model, and the UTW driving frequency, as well as the radius of the sensing circle on the SSS directivity are discussed. The FE results show that the directivity pattern of the second harmonic radiated by the SSS is closely associated with the microcrack orientation, through which the microcrack orientation can be characterized without requiring a baseline signal. It is also found that the SSS directivity varies sensitively with the driving frequency of the incident UTW, while it is insensitive to the stiffness difference between the compressive and tensile phases in the bilinear stress–strain model and the radius of the sensing circle. The results obtained here demonstrate that the orientation of a microcrack can be characterized using the directivity of the SSS induced by the interaction between the incident UTW and the microcrack.

## 1. Introduction

It is well known that even a microcrack may induce a critical threat to the safety and durability of engineering structures [1]. Without timely awareness and subsequent remedial actions, a microcrack may potentially lead to some tragic consequences such as incurring failure of the structures in service. The existence and growth of a microcrack are almost inevitable in engineering structures. It has been found that the orientation of a microcrack has a significant effect on the remaining life of mechanical components [2], claiming the necessity and imminence of developing effective methods capable of characterizing that. However, it is challenging to monitor and characterize the orientation of a microcrack using the conventional linear ultrasonic technique, because the linear acoustic characteristics (such as time of flight (ToF) [3] and transmission and reflection [4,5]) are not sufficiently sensitive to the features of a microcrack including its orientation.

In the past decades, due to the fact that the harmonic generated by primary ultrasonic wave propagation can carry microstructural information of materials, the nonlinear ultrasonic technique has received great attention in nondestructive evaluation (NDE) for its high sensitivity to microdamage and has been proven to be an effective potential for microdamage detection and evaluation [6,7,8]. Generally, the nonlinearity of ultrasonic wave propagation in an elastic solid with microcrack comes from three main sources: the elastic nonlinearity of material [9], the geometric nonlinearity [10], and the contact acoustic nonlinearity (CAN) (or the boundary nonlinearity of contact) [11]. In the field of NDE, there are mainly five techniques for nonlinear ultrasonic detection: vibration acoustic modulation [12], nonlinear wave modulation spectroscopy technique [13,14], nonlinear resonance technique [15], second-harmonic generation [16] and wave-mixing technique [17,18]. The second-harmonic generation based technique has already been studied numerically, analytically, and experimentally to detect the fatigue damage [19,20,21]. So far, in addition to fatigue damage, it has also been used to detect other damage, such as creep [22], thermal aging [23] and radiation damage [24].

For characterizing the features of a microcrack, an appropriate theoretical model describing the nonlinear interaction between the ultrasonic wave and the microcrack is needed. Until now, quite a few theoretical models have been established, such as the bilinear stress–strain model describing the effect of CAN, the surface contact model and hysteresis behavior model, as reviewed by Broda et al. [25]. The bilinear stress–strain model of CAN was mentioned and numerically verified by Friswell et al. [26]. Solodov et al. explained the nonlinearity mechanism of the interaction between a microcrack and an incident ultrasonic wave [11]. In the tensile phase of incident ultrasonic wave impinging on the microcrack, the microcrack is open and the local stiffness is reduced, while the local stiffness is the same as that of the intact solid in the compressive phase. Due to the fact that the model of CAN is relatively intuitive, it has been widely used in analyzing the nonlinear interaction between ultrasonic waves and microcracks [27,28,29]. When an incident ultrasonic wave interacts with a microcrack, the former will be modulated, resulting in a minor waveform distortion without changing the basic synchronism between the incident ultrasonic wave and its response signal. Moreover, the dynamical analysis of a microcracked bar characterized by a uniform distribution of fibers and cracks has been performed [30], through which some useful hints can be furnished to interpret the response of one-dimensional structures with widespread damage.

Generally, information associated with a microcrack may not be efficiently extracted from the response signal to an incident ultrasonic wave in the absence of prior knowledge of its orientation or shape. Thus, it is essential to build a relationship between the microcrack orientation and the response signal to the incident ultrasonic wave modulated by the microcrack. So far, some researchers have built the relationship between the microcrack orientation versus its principal direction and the nonlinear response signal of primary ultrasonic waves. Lv et al. proposed a non-collinear shear wave mixing technique for the evaluation of fatigue crack orientation [31], where the shape of the fatigue crack is assumed to be elliptical. Wang et al. explored the interaction between the probing guided ultrasonic waves and a nonlinear scatterer (e.g., ‘breathing’ fatigue crack) from analytical, simulation, and experimental perspectives, and ascertained the principal orientation of a fatigue crack [28]. Blanloeuil et al. provided the near-field solution for the interaction between an in-plane elastic wave and a closed crack with different orientations using a finite element (FE) approach, yielding the directivity patterns for all linear and nonlinear components of the scattered waves [32]. Zhang et al. developed a FE model to compute the scattering coefficient matrix for arbitrarily shaped defects, where the orientation of a defect was deduced from the location of the maximum scattering coefficient if it lay within the range of probing angles [33]. Obviously, knowledge of the microcrack orientation-related wave scattering phenomena can be beneficial to quantitative evaluation of microcrack severity, which is a crucial but unsolved problem in practical engineering applications. As an effective candidate, second-harmonic generation based approach can be used to realize the identification of the microcrack in an early stage, and to further assess the microcrack orientation in a quantitative manner, which has been one of the most common objectives of the NDE communities [28,32,34]. Recently, a primary attempt has been made by studying the nonlinear interaction between an incident ultrasonic longitudinal wave (ULW) and a microcrack based on the model of CAN [35]. Under the modulation of CAN to the incident ULW impinging on the microcrack, the microcrack itself is treated as a secondary sound source (SSS) radiating the second harmonic. Therefore, the second-harmonic directivity of the SSS (i.e., the SSS directivity) is inherently related to the microcrack itself, including its orientation. According to this consideration, the relationship between the SSS directivity and the microcrack orientation can be established. The simulation results obtained indicate that the SSS directivity is highly sensitive to the change of the microcrack orientation. However, there is a drawback that the second harmonic radiated by the SSS will be contaminated by that generated by the interaction between the incident ULW and the inherent elastic nonlinearity of a solid.

It is well known that when an ultrasonic transverse wave (UTW) propagates in a solid, there is no generation of second harmonic due to the elastic nonlinearity of material and the geometric nonlinearity [36]. When an incident UTW rather than ULW propagates and impinges on a microcrack, due to the modulation of CAN to the incident UTW, the microcrack itself still works as an SSS radiating the second harmonic. In such a situation, the second harmonic is only radiated by the SSS and the interference like that occurring in the case of ULW propagation can be completely avoided. Motivated by this consideration, this paper aims at developing a two-dimensional (2D) FE model with the bilinear stress–strain constitutive relation, which can interpret the nonlinear interaction between the incident UTW and the microcrack examined.

With the FE model established, the generation of a second harmonic can be depicted explicitly, and a quantitative relationship between the microcrack orientation and the directivity of second harmonic radiated by the SSS can be evaluated. This paper is organized as follows: theoretical fundamentals of the CAN and bilinear stress–strain model are presented in Section 2. In this section, the bilinear stress–strain model is explained. The physical mechanism of the CAN for the nonlinear interaction of the incident UTW and the microcrack is presented, and then a 2D FE model based on the CAN is built. In Section 3, an FE simulation is conducted, and the relationship between the SSS directivity and the microcrack orientation is discussed. Meanwhile, the effects of the stiffness difference in the bilinear stress–strain model and the UTW driving frequency on the SSS directivity, as well as the sound field induced by the SSS, are investigated. Finally, conclusions are drawn in Section 4.

## 2. Theoretical Fundamentals

### 2.1. Bilinear Stress–Strain Model

**Figure 1:**
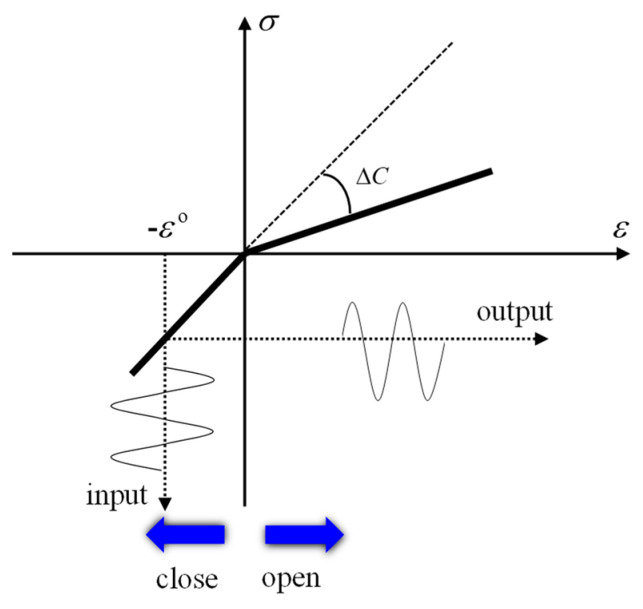
The bilinear constitutive relation between stress and strain.

The bilinear stress–strain model is a frequently-used one describing the effect of the CAN induced by the nonlinear interaction between an ultrasonic wave and a microcrack [11,35,37], where the lack of stiffness symmetry near the microcrack region is assumed. The CAN lies in the fact that the apparent local stiffness around the microcrack region changes under the compressive and tensile phases of the ultrasonic wave impinging on the microcrack. It includes the following two steps: (i) when the compressive phase of the ultrasonic wave occurs at the region of the microcrack, its stiffness is kept unchanged, and the ultrasonic wave penetrates the microcrack like propagating through an intact continuous medium; (ii) when the tensile phase of the ultrasonic wave occurs near the region of the microcrack, the corresponding stiffness is weakened, and then the ultrasonic wave is modulated with a minor waveform distortion, which induces new harmonic components. Both steps jointly give rise to the nonlinear interaction between the ultrasonic wave and the microcrack examined. When the status of the ultrasonic wave propagation near the microcrack turns from the compressive phase to the tensile one, the stress–strain constitutive relation will change, as shown in Figure 1. In its simplest form, such a “bi-modular” area can be simulated by a piece-wise stress–strain constitutive relation [11]:

(1) $$\sigma =C\left[1-H(\varepsilon -\varepsilon^{0})(\Delta C/C)\right]\varepsilon$$

where H(*) is the Heaviside unit step function; $\varepsilon^{0}$ is the initial static contact strain, which defines the so-called “operating point”; $\Delta C=[C-{\left(d\sigma /d\varepsilon \right)}_{\varepsilon >0}]$ is the difference of the elasticity modular between compressive and tensile phases. It is assumed that for the compressive phase there is ε≤0. It is worth mentioning that the amplitudes of the distorted wave are affected by ΔC/C, which will be discussed in Section 3.2.2.

### 2.2. Nonlinear Interaction between the UTW and the Microcrack

**Figure 2:**
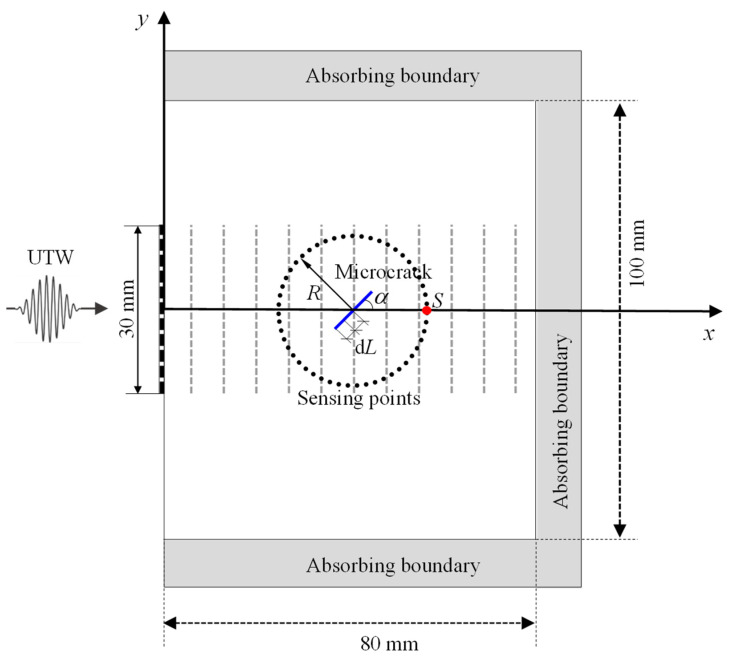
A 2D model of nonlinear interaction between an incident ultrasonic transverse wave (UTW) and a microcrack.

The nonlinear interaction between the UTW and the microcrack can be depicted by the following procedure. As illustrated schematically in Figure 2, we assume that the orientation of the microcrack (denoted by the angle α) is not along the direction of particle vibration of the UTW (i.e., α≠90°). As compared with the wavelength (λ) of the incident UTW, it is assumed that the microcrack is segmented into N segments (λ≫ each segment dL). When the incident UTW impinges on the microcrack, each segment dL of the microcrack successively interacts with the incident UTW, and thus dL itself works as an SSS and radiates a secondary sound field. The total secondary sound field is deemed as the superposition of each secondary sound field radiated by each segment dL. Theoretically, the secondary sound field radiated may include the second and even higher-order harmonics. In this paper, interest focuses on the second harmonic radiated by the SSS, which can completely avoid the interference induced by the elastic nonlinearity of material and the geometric nonlinearity. Due to the fact that the SSS is induced by the interaction between the incident UTW and the microcrack, it must be closely related to the features of the incident UTW (such as amplitude $A_{\mathrm{inc}}$, driving frequency $f_{\mathrm{inc}}$ and phase $\varphi_{\mathrm{inc}}$), as well as that of the microcrack (such as orientation angle α and relative stiffness difference ΔC/C). Therefore, the total second harmonic field radiated by the SSS, denoted by $U_{2f}$, should be a function of $A_{\mathrm{inc}}$, $f_{\mathrm{inc}}$, $\varphi_{\mathrm{inc}}$, α and ΔC/C, i.e.,

(2) $$U_{2f}=f(A_{\mathrm{inc}},f_{\mathrm{inc}},\varphi_{\mathrm{inc}},\alpha ,\Delta C/C)$$

In short, when the incident UTW impinges on the microcrack, due to the effect of CAN, the interaction between them engenders an additional SSS, which will radiate an additional second harmonic field [28,38,39]. In other words, when the incident UTW and the microcrack examined interact with each other, an additional second harmonic field will be radiated, which superimposes on the primary UTW field. Without loss of generality, the primary UTW field is defined as $U_{f}$. Therefore, the total sound field $U_{T}$ is obtained by the following:.

(3) $$U_{T}=U_{f}+U_{2f}$$

When the parameters such as $A_{\mathrm{inc}}$, $f_{\mathrm{inc}}$, $\varphi_{\mathrm{inc}}$, and ΔC/C in Equation (2) are given, it is expected that the feature (e.g., directivity) of $U_{2f}$ is closely related to the orientation angle α (see Figure 2). So, the second harmonic directivity of the SSS can be used to quantitatively characterize the orientation of the microcrack examined. For the case of the UTW incidence, $U_{2f}$ in Equation (3) is only due to the existence of the microcrack and is independent of the inherent elastic nonlinearity of a solid.

### 2.3. Simulation Modeling

It needs to be noted that the present investigation is performed under the precondition that the exact location of the microcrack examined is known in advance. Regarding the method for exactly locating a microcrack, it can be found in [40,41]. Figure 2, meanwhile, shows a 2D model for investigating nonlinear interaction of an incident UTW and a microcrack with orientation angle α, where the solid is assumed to be aluminum with the material parameters listed in Table 1.

As shown in Figure 2, the incident UTW is generated using 0.5 MHz 10 cycles Hanning windowed sinusoidal tone bursts with an amplitude of 100 nm. The origin of the coordinate system is located at the left center of the elastic solid. In the model, the angle α signifies the orientation angle of the microcrack between the principal direction of the microcrack and the positive direction of the *x*-axis, where the length direction of the microcrack is defined as its principal direction. The center of the microcrack with the orientation angle α is assumed to be at the central position located at x = 40 mm and y = 0 mm. The length of the microcrack is set to be 5 mm. The sensing points are distributed along the sensing circle of the specified radius R to extract the desired second harmonic signals radiated by the SSS. The center of the circle is chosen to coincide with the geometrical center of the microcrack in the model.

**Figure 3:**
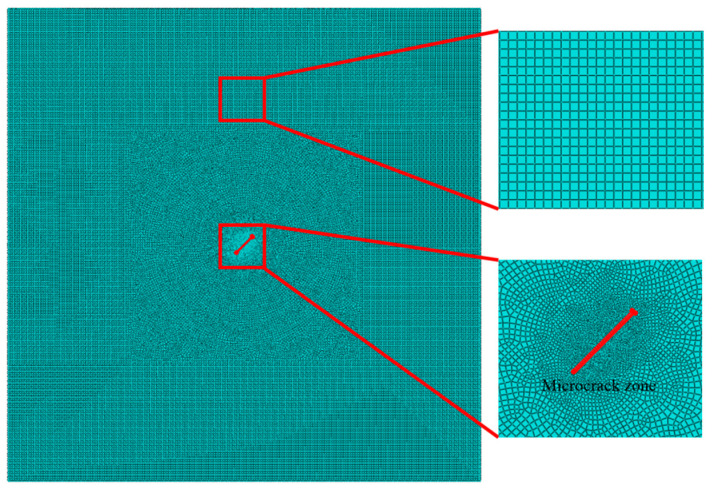
The meshes of local enlargement of the microcrack zone and the remaining region of the model.

A 2D FE model is constructed using the four-node plane strain (CPE4R) elements in software ABAQUS (Version 6.14, Dassault Systèmes Simulia Corp., Providence, RI, USA), where a VUMAT subroutine defining the bilinear stress–strain constitutive relation is implemented to update the stress tensor at every time step. Two-layer meshes are introduced to simulate the existence of the microcrack. The meshes of local enlargement of the microcrack zone and the remaining region are shown in Figure 3. Taking the strain ε as the criterion, it is considered to be positive or negative in tensile or compressive phase, respectively. Mathematically, the stiffness of the two layer meshes is weakened as ε>0 while it is kept unchanged as ε≤0.

Spatial discretization is essential in the FE simulation. The appropriate grid cell edges length ($L_{e}$) and the integration time step (Δt) are adopted, which are based on the minimum wavelength ($\lambda_{\min}$) and the maximum frequency ($f_{\max}$) of the second harmonic radiated by the SSS. The maximum acceptable element size and time step are calculated according to the following equations [42]:

(4) $$L_{e}\leq \frac{\lambda_{\min}}{20},\Delta t\leq \frac{1}{20f_{\max}}$$

The minimal element is 0.31 mm and the smallest time step is $8\times 10^{-9}$ s based on Equation (4). To ensure the computational efficiency and accuracy of FE simulations, the grid cell edge length is selected as 0.15 mm and the lengths of the two-layer meshes modeling the microcrack itself are set to be 0.1 mm (see Figure 3). At the same time, a time step of $5\times 10^{-9}$ s is chosen.

## 3. Simulation Results and Discussions

### 3.1. Analysis of the Second Harmonic Radiated by the SSS

**Figure 4:**
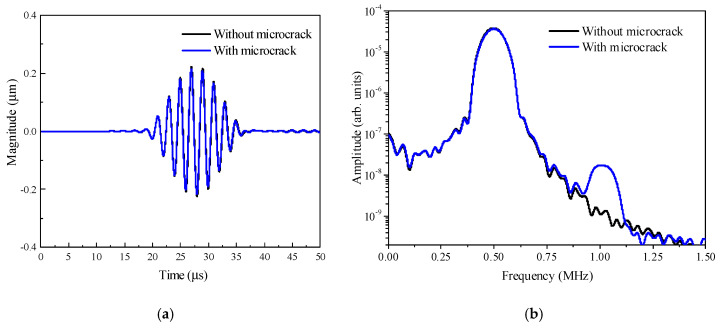
The time-domain signals (**a**) and the corresponding amplitude–frequency curves (**b**) with and without microcrack.

The nonlinear effect induced by the interaction between the incident UTW and the microcrack examined is investigated in this section. Two simulations are considered: the first one is with the intact material, while the second one is with the material with a microcrack like that shown in Figure 2. Figure 4a shows two typical time-domain signals of the mechanical displacements along the vertical direction, which are acquired at the sensing point *S* (60.0, 0.0) mm for the two simulations (see Figure 2). As can be seen from Figure 4a, it is difficult to directly discern the difference between the two time-domain signals. In order to effectively discern the possible difference between them, the corresponding amplitude-frequency curves are calculated and shown in Figure 4b in log scale. Comparing with the two amplitude–frequency curves shown in Figure 4b, the amplitudes at the fundamental frequency are almost kept the same, indicating that it is difficult to identify the existence of the microcrack through the signals at the fundamental frequency. However, it can identify the existence of the microcrack through the difference at the corresponding double frequency.

### 3.2. Directivity Analysis of the SSS

The orientation of the microcrack is generally unknown in engineering structures [31]. The previous analyses indicate that the directivity of the second harmonic radiated by the SSS can be used to characterize the microcrack orientation. Here, the processing procedure for acquiring the SSS directivity is illustrated as follows:

- Step 1: Preprocess the time-domain signals acquired at the sensing circle shown in Figure 2 with Hanning-window modulation;
- Step 2: Calculate the amplitude–frequency curves of the time-domain signals preprocessed;
- Step 3: Extract the peak values of the second harmonic desired, and normalize them by the maximum peak value of the second harmonic acquired at the sensing circle;
- Step 4: Transform the normalized peak values of second harmonics from the Cartesian coordinate system into the polar coordinate system;
- Step 5: Plot the normalized peak values in the polar coordinate system and then obtain the directivity of the SSS.

According to the above processing procedure, some numerical simulations are conducted with different microcrack orientations to investigate the directivity of the second harmonic radiated by the SSS. In addition, the effects of stiffness difference between the compressive and tensile phases in the bilinear stress–strain model, as well as the UTW driving frequency, on the SSS directivity are also investigated.

#### 3.2.1. Effects of the Microcrack Orientation on the Directivity of the SSS

**Figure 5:**
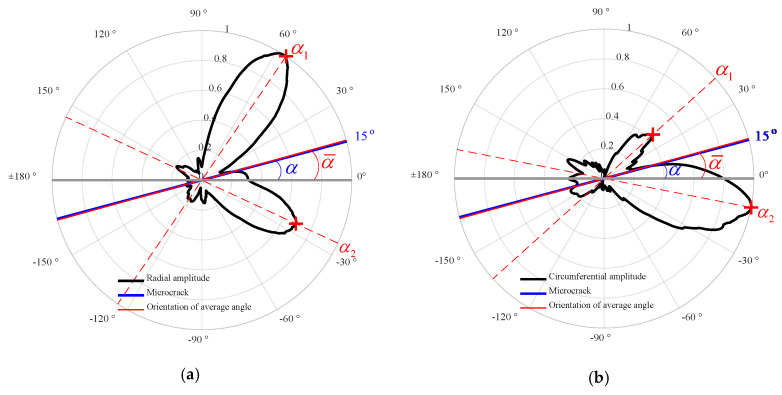

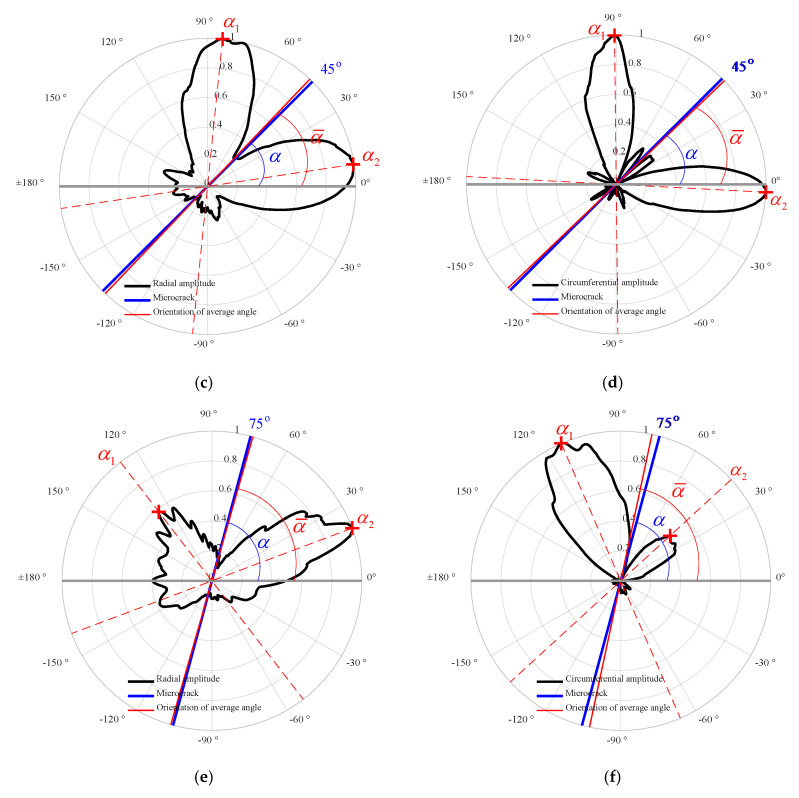
Normalized directivity of the radial (**a**,**c**,**e**) and circumferential (**b**,**d**,**f**) second harmonic amplitudes for several microcrack orientations.

When the incident UTW impinges on the microcrack and the parameters such as $A_{\mathrm{inc}}$, $f_{\mathrm{inc}}$, $\varphi_{\mathrm{inc}}$, and ΔC/C in Equation (2) are given, it is expected that the feature (e.g., directivity) of $U_{2f}$ is closely related to the orientation angle α (see Figure 2). Therefore, the directivity of the second harmonic radiated by the SSS is closely associated with the orientation angle α. In order to investigate such an issue, a case study of the incident UTW impinging on the microcrack with different orientation angles is conducted. The FE simulations for different microcrack orientation angles (15°, 45° and 75°) are carried out to analyze the relationship between the microcrack orientation and the SSS directivity. The horizontal and vertical displacement components in the Cartesian coordinate system are recorded at a set of sensing points of the circle with radius *R* = 20 mm, as shown in Figure 2. The plots of the SSS directivity with several microcrack orientations are shown in Figure 5, where the black solid line represents the amplitudes of the second harmonic acquired from the sensing points of the circle (*R* = 20 mm), and the blue solid line represents the microcrack with a length of 5 mm. Figure 5a,c,e and Figure 5b,d,f correspond, respectively, to the normalized radial and circumferential displacement amplitudes under the polar coordinate system. As can be seen, the directivity of the SSS changes sensitively with respect to the microcrack orientation. By observing the regularity of the SSS directivity with respect to the microcrack orientation, an average angle $\bar{\alpha }$ (i.e., $\bar{\alpha }=(\alpha_{1}+\alpha_{2})/2$, see Figure 5) of the two maximal values (indicated with two red plus sign) distributing on both sides of the microcrack is defined, which matches well with the microcrack orientation angle α so that it can be used as the basis for determining α. Therefore, the plot of the directivity of the second harmonic radiated by the SSS has the potential for detecting the existence of the microcrack and characterizing its orientation with the average angle $\bar{\alpha }$.

#### 3.2.2. Effects of the Stiffness Difference on the Directivity of the SSS

**Figure 6:**
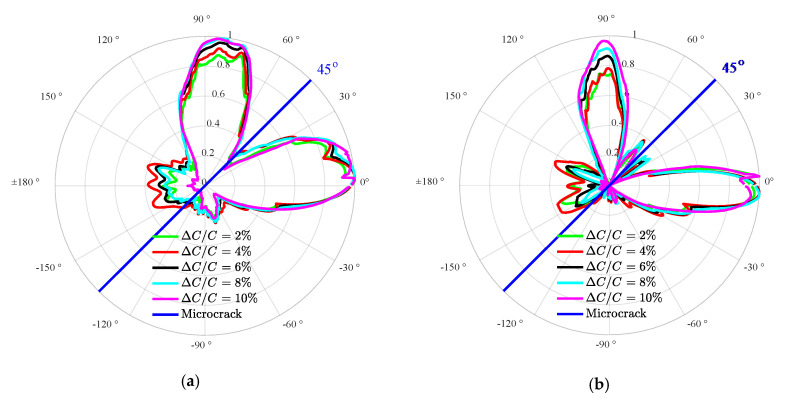
Normalized directivity of the radial (**a**) and circumferential (**b**) second harmonic amplitudes under different stiffness difference ΔC/C (from 2% to 10%).

The stiffness of material is an essential factor in the nonlinear behavior of damage (e.g., microcrack) [43]. In order to further analyze the effects of the stiffness of the microcrack influenced zone on the directivity of the second harmonic radiated by the SSS, the FE simulations with different relative differences of elasticity modulus (i.e., ΔC/C) changing from 2% to 10% with an increment of 2% between the compressive and tensile phases are carried out. The scenario of the orientation angle α is used as a representative for investigating the effects of the stiffness difference on the SSS directivity. A set of the sensing points collecting the response signals are located along the circle of radius *R* = 20 mm. The second harmonic signals are extracted from the sensing points for analyzing the effects of the stiffness difference on the SSS directivity. The driving frequency of the incident UTW is set to be 0.5 MHz. As shown in Figure 6, the displacement amplitudes are normalized with respect to the corresponding maximal peak value of the sensing points. Figure 6a,b show that five different stiffness differences exhibit good coincidence of the normalized directivity of the second harmonic in radial and circumferential displacement components, respectively.

**Figure 7:**
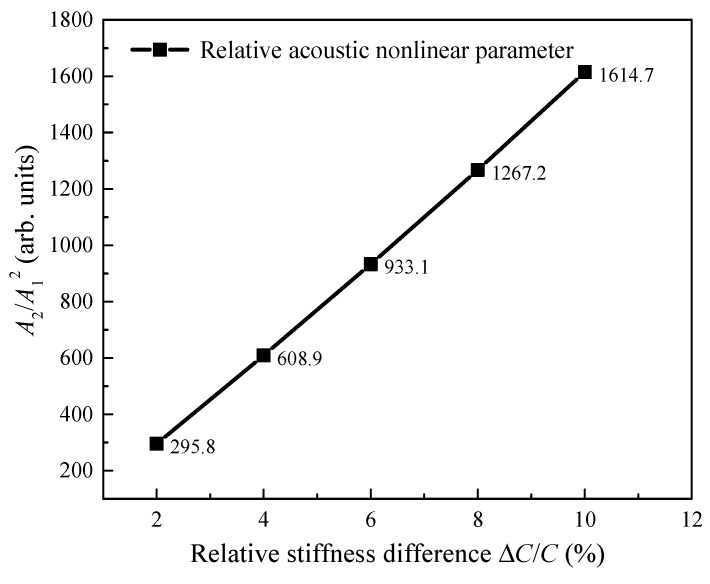
Relative acoustic nonlinear parameter at a sensing point (47.7, 18.5) mm versus the relative stiffness difference (from 2% to 10%).

The relative acoustic nonlinearity parameter ($A_{2}/A_{1}^{2}$) [25] is introduced to intuitively exhibit the relationship between the relative stiffness difference and the second harmonic radiated by the SSS, where $A_{1}$ and $A_{2}$ are the amplitudes of $U_{T}$ and $U_{2f}$ in Equation (3), respectively. Figure 7 shows the relative acoustic nonlinearity parameter ($A_{2}/A_{1}^{2}$) at an arbitrary sensing point (47.7, 18.5) mm versus the stiffness difference (from 2% to 10%). Clearly, the relative acoustic nonlinearity parameter increases linearly with the relative stiffness difference. From Figure 6 and Figure 7, it can be seen that the relative stiffness difference only has an influence on the displacement amplitudes of the second harmonic (i.e., $A_{2}/A_{1}^{2}$), while it has almost no influence on the SSS directivity. In other words, when the driving frequency of UTW remains unchanged, the relative stiffness difference barely has an effect on the directivity of the SSS, while it has an influence on the amplitude of the second harmonic radiated by the SSS. The simulation results show that the microcrack orientation can be characterized by the directivity of the second harmonic radiated by the SSS without consideration of the relative stiffness difference of the microcrack influenced zone.

#### 3.2.3. Effects of the UTW Driving Frequency on the Directivity of the SSS

**Figure 8:**
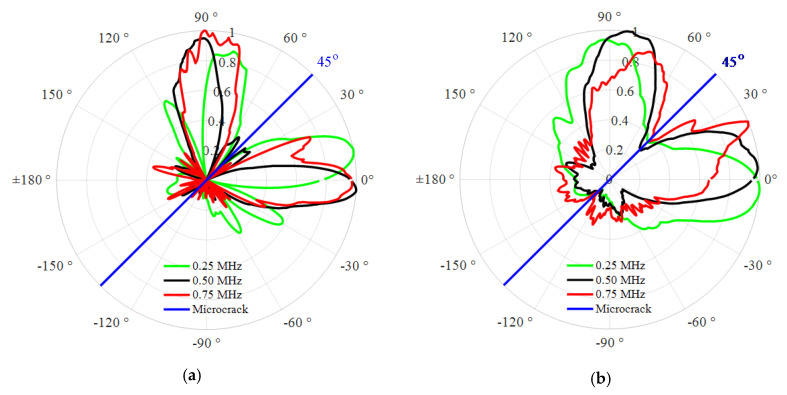
Normalized directivity of the radial (**a**) and circumferential (**b**) second harmonic amplitudes under the UTW driving frequency of 0.25, 0.5 and 0.75 MHz.

The FE simulations are repetitively conducted in a range of frequency from 0.25 to 0.75 MHz with an increment of 0.25 MHz to further investigate the effects of the UTW driving frequency on the SSS directivity. Similarly, the orientation angle α of the microcrack is set to be 45° as a representative for investigating the effects of the UTW driving frequency on the SSS directivity. A set of sensing points collecting the response signals are located along the cycle of the radius *R* = 20 mm. The second harmonic signals are extracted from those sensing points for obtaining the corresponding SSS directivity. Figure 8 shows the normalized directivity under the given UTW driving frequency. The representative UTW driving frequency of 0.25, 0.50 and 0.75 MHz are, respectively, marked in green, black and red lines. The simulation results indicate that the SSS directivities exhibit different patterns under different UTW driving frequencies. This can be attributed to the fact that the incident UTW with different driving frequency possesses different phases and displacements even when it impinges on the same segment dL of the microcrack (see Figure 2). It implies that the UTW driving frequency has an obvious effect on the SSS directivity. However, it can be found that the SSS directivity patterns with different frequencies still approximately distribute on the two sides of the microcrack. Based on this feature, the microcrack orientation can be specified approximately.

#### 3.2.4. Effects of the Radius of the Sensing Circle on the Directivity of the SSS

**Figure 9:**
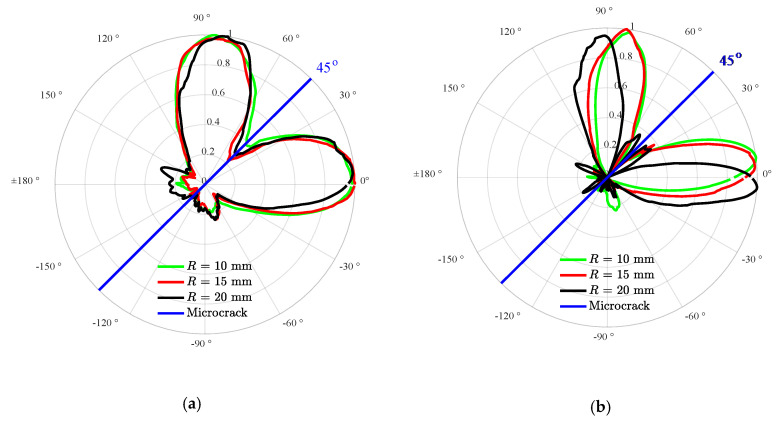
Normalized directivity patterns of the radial (**a**) and circumferential (**b**) second harmonic amplitudes under the sensing circle radii of 10, 15 and 20 mm.

As described in Section 2.2, it is expected that, when the radius *R* of the sensing circle varies, the corresponding change in the SSS directivity will take place. In the FE simulations, the radius *R* of the sensing circle in Figure 2 is set to be 10, 15 and 20 mm to investigate its effect on the directivity of the SSS. The UTW driving frequency is set to be 0.5 MHz, and the relative stiffness difference ΔC/C in Equation (1) is set to be 10%. The simulation results for the orientation angle are shown in Figure 9, where the SSS directivity patterns with the different radius *R* of the sensing circle are presented. It can be seen that the SSS directivity is kept approximately unchanged when the radius *R* of the sensing circle varies within a certain range.

Based on the above FE simulations, it is found that the directivity of the second harmonic radiated by the SSS varies sensitively with the orientation angle α of the microcrack, and that the defined average angle $\bar{\alpha }$ matches well with α. Comparatively speaking, the relative stiffness difference of the microcrack influenced zone has barely any effect on the directivity of the SSS, while it is sensitively affected by the UTW driving frequency. In addition, the radius of the sensing circle has less impact on the directivity pattern of the SSS. Based on these features, the microcrack orientation can be characterized by the directivity of the second harmonic radiated by the SSS.

## 4. Conclusions

This paper aims at quantitatively characterizing the microcrack orientation. Under the modulation of the microcrack via the effect of CAN to the incident UTW impinging on the microcrack examined, the microcrack itself is treated as an SSS radiating the second harmonic. Based on this consideration, the directivity of the second harmonic radiated by the SSS is inherently related to the microcrack itself, including its orientation. It is noteworthy that there is no generation of second harmonic by propagation of primary UTW in a solid and thus the second harmonic generated is only due to the SSS. A 2D FE model is established in conjunction with the bilinear stress–strain constitutive relation. The FE simulation results show that the directivity of the second harmonic radiated by the SSS exhibits the different patterns with respect to the different orientation angles of the microcrack. Furthermore, the FE simulation results also show that the relative stiffness difference of the microcrack influenced zone has barely any effect on the SSS directivity, which only contributes to the magnitude of the second harmonic radiated by the SSS. However, the UTW driving frequency has an obvious effect on the directivity of the SSS. It is also found that the radius of the sensing circle has less impact on the directivity pattern of the SSS. The FE simulation results show that the average angle defined in this paper matches well with the microcrack orientation angle, through which the microcrack orientation can be characterized without requiring a baseline signal.

## Figures and Tables

**Table 1:** Material parameters of the aluminum used in finite element (FE) simulations.

Density (ρ)	Elasticity Modulus (E)	Poisson’s Ratio (ν)	Velocity of Longitudinal Wave $\left(V_{L}\right)$	Velocity of Transverse Wave $\left(V_{T}\right)$
2700 kg/m^3^	69 GPa	0.33	6158 m/s	3103 m/s

## References

[1] Zakar F., Mueller E. (2016). Investigation of a Columbus, Ohio train derailment caused by fractured rail. Case Stud. Eng. Fail. Anal..

[2] Ringsberg J.W. (2001). Life prediction of rolling contact fatigue crack initiation. Int. J. Fatigue.

[3] Xu C., Yang Z., Qiao B. (2019). Traveling distance estimation for dispersive Lamb waves through sparse Bayesian learning strategy. Smart Mater. Struct..

[4] Zhou C., Su Z., Cheng L. (2011). Quantitative evaluation of orientation-specific damage using elastic waves and probability-based diagnostic imaging. Mech. Syst. Signal Process..

[5] Hisao O. (1982). Reflection of elastic waves by an infinitely long ribbon crack. Jpn. J. Appl. Phys..

[6] Chillara V.K., Lissenden C.J. (2015). On some aspects of material behavior relating microstructure and ultrasonic higher harmonic generation. Int. J. Eng. Sci..

[7] Lv H., Jiao J., Meng X., He C., Wu B. (2017). Characterization of nonlinear ultrasonic effects using the dynamic wavelet fingerprint technique. J. Sound Vib..

[8] Wang X., Wang X., Niu D., Hu X. (2018). Application of nonlinear ultrasonic technique to characterize the creep damage in ASME T92 steel welded joints. NDT E Int..

[9] Torello D., Selby N., Kim J.Y., Qu J., Jacobs L.J. (2017). Determination of absolute material nonlinearity with air-coupled ultrasonic receivers. Ultrasonics.

[10] Pau A., Vestroni F. (2019). The role of material and geometric nonlinearities in acoustoelasticity. Wave Motion.

[11] Solodov I.Y., Krohn N., Busse G. (2002). CAN: An example of nonclassical acoustic nonlinearity in solids. Ultrasonics.

[12] Zhang C., He L., Liu S., Yang Q. (2019). A new vibro-acoustic modulation technique for closed crack detection based on electromagnetic loading. Appl. Acoust..

[13] Den A., Johnson P. (2000). Nonlinear Elastic Wave Spectroscopy (NEWS) Techniques to Discern Material Damage, Part I: Nonlinear Wave Modulation Spectroscopy (NWMS). Res. Nondestruct. Eval..

[14] Ogam G., Groby J.P., Ogam E. (2014). A non-linear vibration spectroscopy model for structures with closed cracks. Int. J. Non-Linear Mech..

[15] Maier S., Kim J.Y., Forstenhäusler M., James J., Laurence J. (2018). Noncontact nonlinear resonance ultrasound spectroscopy (NRUS) for small metallic specimens. NDT E Int..

[16] Deng M., Pei J. (2007). Assessment of accumulated fatigue damage in solid plates using nonlinear Lamb wave approach. Appl. Phys. Lett..

[17] Li F., Zhao Y., Cao P. (2018). Mixing of ultrasonic Lamb waves in thin plates with quadratic nonlinearity. Ultrasonics.

[18] Ding X., Zhao Y., Deng M., Shui G., Hu N. (2019). One-way Lamb mixing method in thin plates with randomly distributed microcracks. Int. J. Mech. Sci..

[19] Zhu W., Deng M., Xiang Y. (2016). Second harmonic generation of Lamb wave in numerical perspective. Chin. Phys. Lett..

[20] Li W., Jiang C., Deng M. (2019). Thermal damage assessment of metallic plates using a nonlinear electromagnetic acoustic resonance technique. NDT E Int..

[21] Shui G., Wang Y., Gong F. (2013). Evaluation of plastic damage for metallic materials under tensile load using nonlinear transverse waves. NDT E Int..

[22] Matlack K.H., Kim J.Y., Jacobs L.J., Qu J. (2015). Review of second harmonic generation measurement techniques for material state determination in metals. J. Nondestruct. Eval..

[23] Metya A., Ghosh M., Parida N., Sagar S.P. (2008). Higher harmonic analysis of ultrasonic signal for ageing behaviour study of C-250 grade maraging steel. NDT E Int..

[24] Matlack K., Wall J., Kim J.Y., Qu J., Jacobs L., Viehrig H.W. (2012). Evaluation of radiation damage using nonlinear ultrasound. J. Appl. Phys..

[25] Broda D., Staszewski W., Martowicz A., Uhl T., Silberschmidt V. (2014). Modelling of nonlinear crack–wave interactions for damage detection based on ultrasound—A review. J. Sound. Vib..

[26] Friswell M.I., Penny J.E.T. (2002). Crack modeling for structural health monitoring. Struct. Health Monit..

[27] Wang K., Fan Z., Su Z. (2018). Orienting fatigue cracks using contact acoustic nonlinearity in scattered plate waves. Smart Mater. Struct..

[28] Wang K., Liu M., Su Z., Yuan S., Fan Z. (2018). Analytical insight into “breathing” crack-induced acoustic nonlinearity with an application to quantitative evaluation of contact cracks. Ultrasonics.

[29] Wang K., Li Y., Su Z., Guan R., Lu Y., Yuan S. (2019). Nonlinear aspects of “breathing” crack-disturbed plate waves: 3-D analytical modeling with experimental validation. Int. J. Mech. Sci..

[30] Settimi V., Trovalusci P., Rega G. (2019). Dynamical properties of a composite microcracked bar based on a generalized continuum formulation. Continu. Mech. Therm..

[31] Lv H., Jiao J., Wu B. (2018). Evaluation of fatigue crack orientation using non-collinear shear wave mixing method. J. Nondestruct. Eval..

[32] Blanloeuil P., Meziane A., Norris A.N. (2016). Analytical extension of finite element solution for computing the nonlinear far field of ultrasonic waves scattered by a closed crack. Wave Motion.

[33] Zhang J., Drinkwater B.W., Wilcox P.D. (2008). Defect characterization using an ultrasonic array to measure the scattering coefficient matrix. IEEE Trans. Ultrason. Ferroelectr. Freq. Control.

[34] Blanloeuil P., Meziane A., Bacon C. (2014). Numerical study of nonlinear interaction between a crack and elastic waves under an oblique incidence. Wave Motion.

[35] Wang J., Liu L., Hu N., Deng M. (2019). Characterization of microcrack’s orientation using secondary acoustic source directivity induced by an incident ultrasonic longitudinal wave. J. Shanxi Norm. Univ. (Nat. Sci. Ed.).

[36] Norris A.N. (1991). Symmetry conditions for third order elastic moduli and implications in nonlinear wave theory. J. Elast..

[37] Zheng Y., Maev R.G., Solodov I.Y. (1999). Nonlinear acoustic applications for material characterization: A review. Can. J. Phys..

[38] Pruell C., Kim J., Qu J., Jacobs L.J. (2009). Evaluation of fatigue damage using nonlinear guided waves. Smart Mater. Struct..

[39] Zhou C., Hong M., Su Z., Wang Q., Cheng L. (2013). Evaluation of fatigue cracks using nonlinearities of acousto-ultrasonic waves acquired by an active sensor network. Smart Mater. Struct..

[40] Liu X., Bo L., Yang K., Liu Y., Zhao Y., Zhang J., Hu N., Deng M. (2018). Locating and imaging contact delamination based on chaotic detection of nonlinear Lamb waves. Mech. Syst. Signal Process..

[41] Hong M., Su Z., Lu Y., Sohn H., Qing X. (2015). Locating fatigue damage using temporal signal features of nonlinear Lamb waves. Mech. Syst. Signal Process..

[42] Shen Y., Giurgiutiu V. (2013). Predictive modeling of nonlinear wave propagation for structural health monitoring with piezoelectric wafer active sensors. J. Intel. Mat. Syst. Struct..

[43] Biwa S., Hiraiwa S., Matsumoto E. (2007). Stiffness evaluation of contacting surfaces by bulk and interface waves. Ultrasonics.

